# A Deep Learning Framework for Gastric Cancer Cell Segmentation with Multi-Scale Attention Mechanisms

**DOI:** 10.3390/bioengineering13070740

**Published:** 2026-06-25

**Authors:** Xinyu Zhao, Jin Liu, Jingru Zhang, Damin Ding, Haima Yang, Bo Huang

**Affiliations:** 1 School of Electronic and Electrical Engineering, Shanghai University of Engineering Science, Shanghai 201620, China; m325124536@sues.edu.cn (X.Z.); dingdm@sues.edu.cn (D.D.); huangbosues@sues.edu.cn (B.H.); 2 Centre for Instructional Technology and Multimedia, Universiti Sains Malaysia (USM), Gelugor 11700, Pulau Pinang, Malaysia; 3 School of Optical-Electrical and Computer Engineering, University of Shanghai for Science and Technology, Shanghai 200093, China; snowyhm@usst.edu.cn

**Keywords:** channel attention, deep learning, feature fusion, gastrointestinal

## Abstract

The accurate segmentation of gastric cancer cells is important in pathology for diagnosing and detecting diseases early. However, current approaches still suffer from limitations such as expensive annotation, fuzzy lesion boundaries, and weak feature expression. In order to solve these problems, we present MSAF-Net, a novel U-Net framework optimized both architecturally and in terms of the loss function. In particular, we incorporate a Multi-scale Dilated Pooling Fusion Block into the encoder stage to achieve enhanced interaction of multi-paths and thus improve features’ diversity and boundary sensitivity. We also introduce a Dual-Channel Attention Block in place of traditional convolution block in the decoder stage to restore better details and reconstruct the fuzzy boundaries. Meanwhile, a Diagonal Mahalanobis Consistency Loss is incorporated into our framework to facilitate class compactness. Experiments performed on the SEED-Gastric Carcinoma Stage 1 dataset show that the designed algorithm can reach 0.776 in Dice score and 0.821 in Accuracy, which outperforms the baseline method U-Net. It is clear that these results have shown the effectiveness and robustness of our proposed approach. The introduced algorithm allows for more precise quantification of gastric cancer cell morphology.

## 1. Introduction

Gastric cancer is one of the most common malignant tumors in China [1]. It ranks fifth among all malignant tumors in terms of incidence and mortality rates, posing a substantial burden on public health [2]. However, because the symptoms of gastric cancer are often atypical during its early stage, most patients are diagnosed during its advanced stage and treatment outcomes are poor. Diagnosis for early gastric cancer lesions is of great practical significance [3,4,5]. The pathological diagnosis is the gold standard for diagnosing cancer [6]. Due to the shortage of pathologists in China and unequal medical resources in different areas, especially remote areas, pathological diagnosis faces huge challenges [7,8,9]. Under such conditions, cell segmentation with high accuracy by using a computer-aided method has become an important way to improve the efficiency of pathological diagnosis [10].

Deep learning has demonstrated strong potential in pathology image analysis. Many segmentation models have been developed to assist pathologists in identifying lesion regions more efficiently. Among them, the U-Net model with its characteristic encoder–decoder architecture has become the benchmark model for medical image segmentation tasks. However, the case images in gastric cancer usually present blurred boundaries and homogeneous coloration due to uneven staining. Lesion regions occupy only a small fraction of the total image area, and prominent features are easily overwhelmed by background information. During downsampling in U-Net, a lot of feature information is discarded, which restricts this model’s capability of fully learning multi-scale lesion details. Its skip connections directly concatenate the shallow and deep features. This results in a number of problems, such as insufficient feature fusion and missed small target regions. U-Net and its derivative networks require high-quality data. However, the annotation of medical images requires experienced pathologists, so the labeling process is usually costly and inefficient. The limited availability of publicly annotated gastric cancer pathology datasets further adds to these challenges. In addition, models under limited data tend to overfit, resulting in poor generalization capability. Therefore, their clinical application faces challenges since it is hard to adapt to the variety of pathological image features.

Many studies have attempted to improve U-Net for medical image segmentation. Representative examples include U-Net++, DeepLabv3+, CFANet, CSCAU-Net, and TransUNet. These methods introduce different strategies, such as enhanced skip connections, multi-scale feature extraction, attention mechanisms, and Transformer structures, to improve feature learning.

These methods improve segmentation performance in medical images. However, gastric cancer pathology images remain challenging. Lesion regions usually occupy only a small part of the image and are easily affected by surrounding tissue structures. During feature extraction, useful information may be weakened after multiple downsampling operations. In addition, uneven staining and tissue heterogeneity increase the similarity between lesion and background regions, making accurate segmentation more difficult. Attention-based and Transformer-based methods can improve feature learning, but they often require more training data. Most existing methods are also trained in a fully supervised manner. Since pixel-level annotation of pathology images is labor-intensive and expensive, the practical application of these methods is still limited. Therefore, developing a framework that can utilize limited labeled data while enhancing lesion feature representation remains necessary.

Obtaining pixel-level annotations for gastric cancer pathology images is labor-intensive and requires extensive expert involvement. Consequently, a large number of pathology images remain unlabeled in clinical practice. Semi-supervised learning provides an effective solution by leveraging a limited amount of labeled data together with abundant unlabeled samples, thereby reducing annotation costs while maintaining model performance.

Motivated by these challenges, this study proposes a Mean Teacher-based semi-supervised segmentation framework for gastric cancer cell segmentation. The proposed framework integrates multi-scale attention mechanisms and a Mahalanobis distance-based loss function to enrich feature representation and segmentation accuracy.

Based on this, an improved U-Net model is proposed for the segmentation of gastric cancer cells using semi-supervised learning. The contributions are as follows:

1. We propose a semi-supervised framework, MSAF-Net, to effectively leverage unlabeled data to improve segmentation performance.
2. We introduce a Multi-scale Dilated Pooling Fusion (MDPF) module to capture multi-scale features and preserve edge information.
3. We employ a Dual-Channel Attention (DCA) module to improve spatial detail recovery during upsampling and enhance boundary reconstruction.
4. We design a Diagonal Covariance Matrix Distance-based (DMC) loss function to increase feature discrimination and maintain stable training.

## 2. Related Work

Prewitt et al. [11] reported the scanning and acquisition of microscopic fields in traditional blood smears and converted the obtained images into optical density matrices. This formed the foundation of digital pathology analysis. With the rapid development of artificial intelligence, pathology image analysis has developed from expert recognition, via machine learning, to deep learning. Early pathology image analysis mainly depended on handcrafted features and traditional image processing methods. Machine learning techniques were later introduced to improve analytical performance. More recently, deep learning has become the dominant approach for pathology image analysis. The most widely applied deep learning model in current use is the CNN architecture [12]. This architecture processes images through multiple layers of convolutional and pooling operations that allow efficient extraction of key feature information and the accomplishment of tasks such as classification, segmentation, and detection. The U-Net++ architecture [13] embeds a series of convolutional layers in its skipping connections to enrich the stability of training and generalization capability. DeepLabv3+ [14] adopts a lightweight decoder with dilated convolutions to combine high semantic level features with low-level spatial details for improved edge localization accuracy. ResNet [15] resolves difficulties in the training process of very deep networks by using a residual learning method that leads to stronger feature representation capabilities. CFANet [16] performs image segmentation with improved global semantic and local detail representation using a feature aggregation module based on multi-scale contextual feature fusion. PraNet [17] proposes a parallel reverse attention structure that enhances boundary sensitivity and tackles issues of low contrast and occlusion frequently found in medical images. CSCAU-Net [18] employs a cross-scale interactive attention mechanism enabling the network to highlight both local edge information and global contextual information. This approach enhances robustness and finer segmentation capabilities in medical images. CNN-based methods have achieved encouraging results in medical image segmentation. They are effective in learning local feature representations. Attention mechanisms further improve feature selection and information aggregation. However, complex tissue backgrounds and limited annotated data still affect segmentation performance in pathology images. These factors remain important challenges for current segmentation models. Transformer-based methods have been introduced into medical image segmentation. TransUNet [19] combines Transformer and U-Net architectures. It uses both global context and local features for segmentation. Large amounts of training data are usually required.Swin-Unet [20] introduces a hierarchical Transformer architecture and improves the utilization of global contextual information. UCTransNet [21] enhances feature interaction between encoder and decoder stages through Transformer-based channel modeling, which improves feature transmission during segmentation.

These methods have improved segmentation performance in medical image analysis. Several challenges still exist in gastric cancer pathology images. Lesion regions usually occupy only a small part of the image. Surrounding tissue structures may interfere with feature extraction. Useful information may be weakened after repeated downsampling operations. Uneven staining and tissue heterogeneity further increase segmentation difficulty. Most existing methods rely on fully supervised learning and require a large number of pixel-level annotations. The acquisition of such annotations is expensive and time-consuming. These factors limit the practical application of many segmentation models.

## 3. Methods

**Figure 1:**
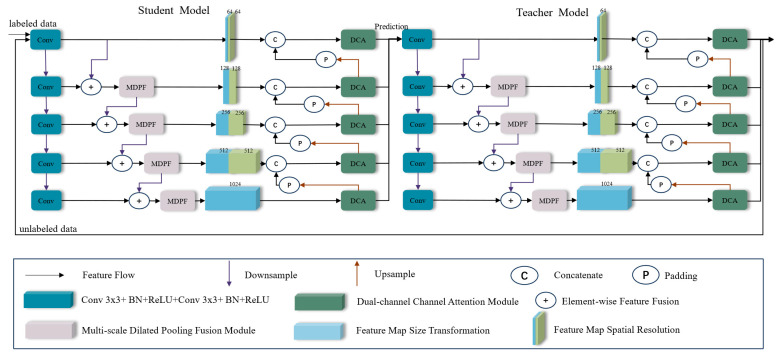
Architecture of MSAF-Net.

The network model proposed in this study is illustrated in Figure 1. The MSAF-Net follows the Mean Teacher architecture, which consists of a student network and a teacher network that have identical structures. The parameters of the student structure are updated by backpropagation, while the teacher model parameters are updated using the exponential moving average of the student parameters. This strategy ensures that during training, the feature representation of the teacher model remains smoother and more stable. During the encoder phase, in order to enhance the capability of multi-scale feature expression, the MDPF module is introduced after each down-sampling layer in MSAF-Net. The MDPF module fuses features of the same scale sequentially in different paths and enhances the representation of local texture and boundary information. During decoding, a DCA module is introduced to further enhance the model’s ability to fuse semantic information with low-level detailed features during up-scaling. By incorporating a multi-level design that employs deep convolution decomposition, multi-path attention reweighting, and feature reconstruction, the proposed module facilitates feature recovery while maintaining computational efficiency.

**Figure 2:**
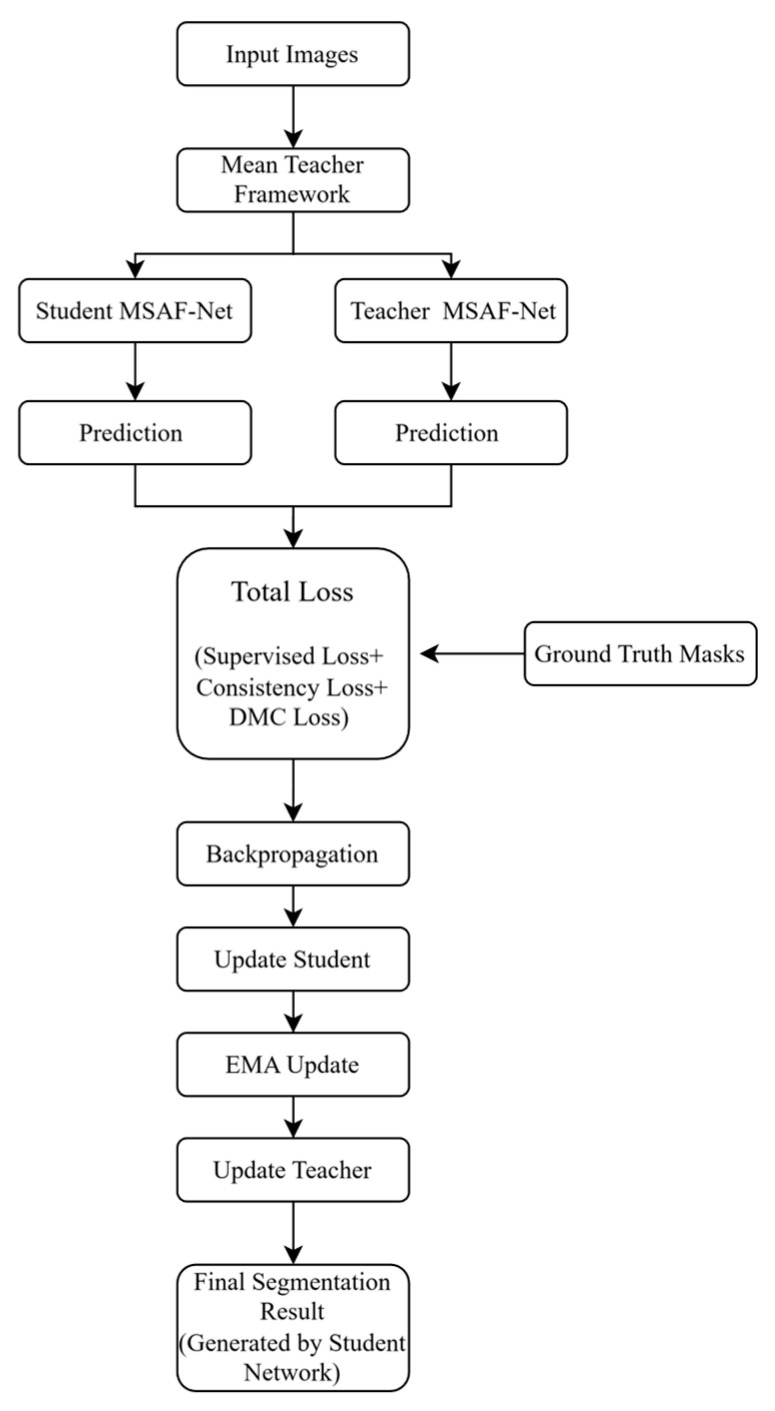
Overall training workflow of the proposed MSAF-Net.

Figure 2 shows the training workflow of the proposed MSAF-Net. The framework contains a student network and a teacher network. Both networks share the same architecture. The proposed MDPF module is used to capture multi-scale features, while the DCA module is used to improve feature fusion during decoding.

During training, the input images are processed by both networks. The student and teacher networks generate segmentation predictions. Ground-truth masks are used to calculate the supervised loss. Consistency loss is introduced to constrain the predictions of the two networks. The proposed DMC loss is added to improve feature discrimination. These loss terms are combined to form the total loss.

The student network is updated through backpropagation. The teacher network is updated using an exponential moving average (EMA) strategy. After training, only the student network is retained for inference and produces the final segmentation results.

### 3.1. Multi-Scale Dilated Pooling Fusion Module

In the traditional process of downsampling, the semantic information keeps becoming more enriched. However, convolutions and pooling tend to degrade feature information, particularly those of vital edges and texture of gastric cancer cells [22]. We propose an adaptive multi-scale feature fusion module in the second path of the encoder to improve the capability of representing local details during the downsampling process and add multi-scale contextual information.

**Figure 3:**
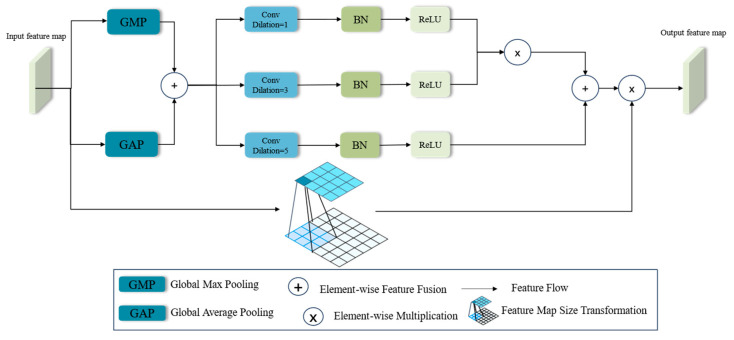
Structure of the Multi-scale Dilated Pooling Fusion (MDPF) module.

As illustrated in Figure 3, this module first conducts dual-branch compression on the input features with average pooling and max pooling to retain background trends and salient texture information, respectively. Then, the pooled features are fed into three 3 × 3 convolutional branches with different dilation rates to capture multi-scale features from fine textures to global patterns.

To further strengthen the relationships between multiscale features, the module performs pointwise multiplication of small- and medium-scale branch features, adding these to the large-scale branch features. This achieves cross-scale feature enhancement and compensation. Finally, it bilinearly interpolates the enhanced features back to the original spatial resolution and multiplies them pointwise with the input features. It enables the re-calibration of key regions and the suppression of redundant background. The above process can be expressed as:

(1) y=AvgPool(F)+MaxPool(F)

(2) $$z_{1}={\mathrm{Conv}}_{d=1}(y),\;\;\;\;z_{3}={\mathrm{Conv}}_{d=3}(y),\;\;\;\;z_{5}={\mathrm{Conv}}_{d=5}(y)$$

(3) $$f=(z_{1}⊙z_{3})+z_{5}$$

where F denotes the input feature map. y represents the pooled feature representation obtained by combining average pooling and max pooling operations. $z_{1}$, $z_{3}$, and $z_{5}$ denote the multi-scale features extracted using dilated convolutions with dilation rates of 1, 3, and 5, respectively. f represents the fused feature map after cross-scale feature enhancement.

### 3.2. Dual-Channel Attention Module

In the process of decoding, the network progressively restores the extracted multi-scale semantic features to their original resolution while enhancing the expressive power of the key regions [23]. This study proposes a Dual-Channel Attention Module to refine channel features with the preservation of spatial information.

**Figure 4:**
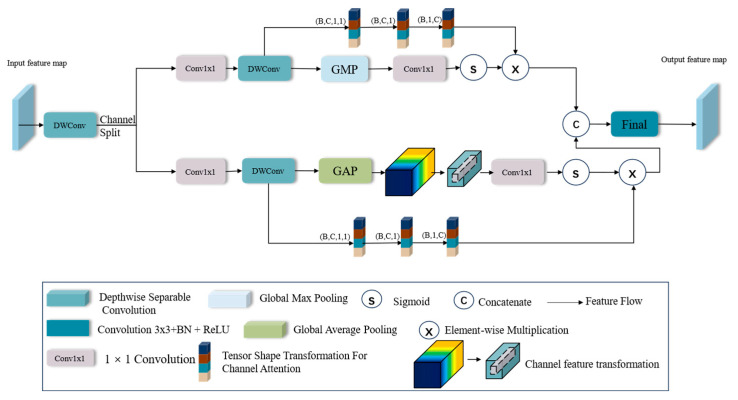
Dual-Channel Attention Module.

As shown in Figure 4, the input features are fed into a DCA module through a depthwise separable convolution, which splits the input channels into two parts. To reduce computational consumption, each part is subjected to channel compression through a 1 × 1 convolution. Then, each feature stream goes through a dedicated channel attention module. One path uses global average pooling-based ECA attention while the other path is based on ECMA attention that uses global max pooling. It adaptively highlights key features at the channel dimension and suppresses redundant information.

These attention-weighted feature streams are then concatenated along the channel dimension. Through a final 3 × 3 convolution, batch normalization, and ReLU activation, feature fusion and dimension matching are accomplished. This module recovers spatial resolution while taking advantage of attention mechanisms that elevate sensitivity toward target regions, hence providing high-quality feature representations for further processing. The whole process could be expressed as:

(4) $$D=\mathrm{DepthwiseConv}(F_{\mathrm{in}})$$

(5) $$D_{1},D_{2}=\mathrm{SplitChannels}(D)$$

(6) $${\hat{D}}_{1}=\mathrm{ECA}(D_{1}),\;\;\;\;{\hat{D}}_{2}=\mathrm{ECMA}(D_{2})$$

(7) $$D_{\mathrm{cat}}=\mathrm{Concat}({\hat{D}}_{1},{\hat{D}}_{2})$$

(8) $$D_{\mathrm{out}}=\mathrm{ConvBNReLU}(D_{\mathrm{cat}})$$

where $F_{\mathrm{in}}$ denotes the input feature map. D represents the feature map after depthwise convolution. $D_{1}$ and $D_{2}$ are the two channel groups obtained by channel splitting. ${\hat{D}}_{1}$ and ${\hat{D}}_{2}$ denote the attention-refined feature maps generated by the two attention branches. The concatenated feature map is denoted by $D_{\mathrm{cat}}$, and $D_{\mathrm{out}}$ represents the final output feature map of the DCA module.

### 3.3. Diagonal Mahalanobis Consistency Loss

In this work, a Mahalanobis distance-based center loss function is added to make the model sensitive to category centers and suppress background interference. Here the loss considers only the diagonal covariance and provides stable constraints for multi-class features by comparing the predicted probability distribution with the category centers of the true labels.

The feature map is averaged over the spatial dimensions to obtain the predicted category center and the target category center for each category:

(9) $$c_{\mathrm{pred}}^{\left(c\right)}=\frac{1}{HW}\sum_{h=1}^{H}\sum_{w=1}^{W}{\mathrm{pred}}_{c,h,w},\;\;\;\;c_{\mathrm{target}}^{\left(c\right)}=\frac{1}{HW}\sum_{h=1}^{H}\sum_{w=1}^{W}{\mathrm{target}}_{c,h,w}$$

where H and W denote the height and width of the feature map, respectively. ${\mathrm{pred}}_{c,h,w}$ and ${\mathrm{target}}_{c,h,w}$ represent the predicted probability and ground-truth label at spatial location (h,w) for category c. $c_{\mathrm{pred}}^{\left(c\right)}$ and $c_{\mathrm{target}}^{\left(c\right)}$ denote the predicted category center and target category center, respectively.

Then, the Mahalanobis distance is computed by normalizing its variance through inter-category differences:

(10) $$d_{i}=\sum_{c=1}^{C}\frac{{\left(c_{\mathrm{pred}}^{\left(c\right)}-c_{\mathrm{target}}^{\left(c\right)}\right)}^{2}}{\mathrm{Var}(c_{\mathrm{pred}}^{\left(c\right)})+\epsilon }$$

To prevent losses from exploding due to outliers, an upper bound is introduced to limit the distance:

(11) $$L_{\mathrm{mahalanobis}}=\frac{1}{N}\sum_{i=1}^{N}\mathrm{clamp}\left(d_{i},0,{\mathrm{clamp}}_{\max}\right)$$

In particular, the loss function constrains the prediction probabilities of different categories to cluster around the true center in the feature space during training and can enhance the segmentation stability of the model in small-sample and boundary regions. This will result in stronger guidance signals for semi-supervised learning.

### 3.4. Overall Framework and Module Collaboration

MSAF-Net is designed as a unified framework for medical image segmentation. Instead of treating each module separately, the network follows a progressive feature refinement process from encoder to decoder.

In the encoder stage, the Multi-scale Dilated Pooling Fusion (MDPF) module is applied first. It captures contextual information at different scales by combining features from multiple receptive fields. As a result, the spatial representation becomes more complete, which is important for handling blurred boundaries and complex lesion structures.

The MDPF module operates on encoder feature maps before skip connection fusion, while the DCA module is applied to decoder-stage fused features. DCA refines the fused features by modeling channel-wise dependencies. Such enriched representations make channel-wise attention more stable and discriminative. The improvement mainly comes from the fact that spatial context from MDPF provides richer feature distributions, which allows DCA to perform more effective channel-wise selection. It learns to highlight informative channels while suppressing redundant responses, which improves the discriminative ability of feature representations and strengthens lesion localization.

This sequential design ensures that spatial feature enrichment and channel-wise refinement are performed in a continuous pipeline without introducing additional branches. In this way, MDPF first increases feature diversity at multiple scales, and DCA further improves feature selectivity.

The two modules are complementary. Without this combination, the model either lacks sufficient spatial diversity or cannot suppress redundant features. MDPF focuses on extracting rich spatial context, while DCA focuses on filtering and recalibrating features in the channel dimension. Their combination provides a balance between feature completeness and feature discrimination, which is beneficial for complex medical image segmentation tasks.

To further improve segmentation performance, the network is optimized using a combination of BCE loss, Dice loss, and DMC loss. This joint supervision helps the model learn both pixel-level accuracy and structural consistency.

The two modules form a complementary learning process rather than independent components. This combination improves segmentation performance by jointly enhancing spatial representation and channel-wise feature refinement.

The following algorithm summarizes the overall workflow of the proposed method, as shown in Algorithm 1.
**Algorithm 1:** MSAF-Net for Medical Image SegmentationInput: Image I Output: Segmentation map S 1: Initialize student network, teacher network (EMA) 2: Initialize encoder, MDPF module, DCA module, decoder 3: for each training iteration do: 4:         F ← Encoder(I) 5:         f ← MDPF(F) 6:         $D_{\mathrm{out}}$ ← DCA(f) 7:         S ← Decoder($D_{\mathrm{out}}$) 8: Loss computation: 9:        $L=\omega (t)\cdot L_{cons}\;+L_{\mathrm{BCE}}+L_{\mathrm{Dice}}+\lambda_{m}L_{Mahalanobis}$ 10: Backpropagation: 11:        Update student network parameters 12: Teacher update: 13:        θ_teacher ← α · θ_teacher + (1 − α) · θ_student 14: end for 15: Return S

## 4. Experiments and Results

### 4.1. Dataset

The two publicly available datasets related to gastric cancer histopathology were used: SEED-Gastric Carcinoma [24] and Data for HCRF [25]. For fully supervised training, the SEED-Gastric Carcinoma dataset was employed, whereas the Data for HCRF dataset was used for unlabeled data for semi-supervised training.

The SEED-Gastric Carcinoma dataset consists of 1770 pathology images and their corresponding mask images. These mask images were used to demarcate not only the normal but also the pathological regions. All these annotations were performed by experienced pathologists. In the experiments, both datasets contained uninfected slide images. In addition, completely black mask images took part in the mean calculation; it may lead to slightly lower evaluation metrics.

The 600 clinical images in the HCRF dataset do not include masks, so they are used as the source of semi-supervised training.

Following the documentation of the dataset, medical images and their masks from SEED-Gastric Carcinoma were partitioned. The exact details are that 80% of the PNG images in each dataset were used for the training set, and the remaining 20% formed the validation set.

### 4.2. Evaluation Metrics

The experiment employs four evaluation metrics to assess model performance quantitatively. Relevant descriptions are as follows:

Dice: The Dice coefficient is used to measure the overlap.

(12) $$Dice=\sum_{i=1}^{C}\frac{2TP_{i}}{2TP_{i}+FP_{i}+FN_{i}}$$

Accuracy: Proportion of correctly classified pixels by the model against total number of pixels.

(13) $$Accuracy=\frac{TP+TN}{TP+FP+FN+TN}$$

Recall: The proportion of pixels in the target region correctly identified by the model relative to the total number of actual target pixels.

(14) $$Recall=\frac{TP}{TP+FN}$$

MIoU: MIoU is the fraction of the intersection area over the union area between the prediction and ground truth across all classes.

(15) $$MIoU=\frac{1}{k+1}\sum_{i=0}^{k}\frac{TP}{TP+FP+FN}$$

### 4.3. Implementation Details

The experimental platform used in this paper is Ubuntu 20.04. The CPU uses an Intel^®^ Xeon^®^ Platinum 8255C CPU (Intel Corporation, Santa Clara, CA, USA) with a base frequency of 2.50 GHz, while the GPU uses an Nvidia RTX 3090 (NVIDIA Corporation, Santa Clara, CA, USA) with 24 GB of memory capacity. Python 3.6.15 was used to develop the model. The network was implemented with PyTorch 1.10.2 (Meta AI, Menlo Park, CA, USA). CUDA 12.1.0 (NVIDIA Corporation, Santa Clara, CA, USA) was used to speed up GPU computation. For the proposed DMC loss, the numerical stability parameter ϵ was set to 0.001 in Equation (10), and the upper bound parameter clamp_max in Equation (11) was set to 100.0 to prevent extremely large Mahalanobis distance values caused by outliers during training.

In this work, a semi-supervised medical image segmentation model was implemented using the Mean Teacher strategy with PyTorch [26], a deep learning framework. The EMA updates the parameters with a decay factor of 0.995 to ensure that the teacher model is trained to have smoother and more stable representation capabilities.

The overall loss of the student model during training is a weighted sum of supervised loss and consistency loss. For unlabeled data, consistency loss is defined as the KL divergence between the probability distributions of the outputs of the student model and the teacher model. Noise in pseudo labels is suppressed by a confidence filtering mechanism that calculates the consistency term only at pixel positions with high confidence from the teacher prediction. The same augmented unlabeled image is provided to both the Student and Teacher networks during training. The augmentation pipeline consists of random scaling, random cropping, horizontal flipping, rotation, and brightness/contrast adjustment. For unlabeled data, teacher predictions are used to guide consistency learning. Since the quality of pseudo-labels may vary during training, a confidence filtering strategy is adopted. Specifically, pixels with confidence scores greater than 0.3 are selected for consistency supervision, while low-confidence predictions are ignored. This strategy helps reduce the influence of noisy pseudo-labels and improves training stability. The threshold value was determined through preliminary experiments and provided a good balance between pseudo-label reliability and sample utilization. The weight on the consistency loss incorporates a sigmoid ramp-up to avoid model over-reliance on pseudo labels at the start of training.

The optimizer utilizes stochastic gradient descent [27] with an initial learning rate of 0.0001, momentum of 0.9, and a weight decay coefficient of 0.0001. Training uses automatic mixed precision to save GPU memory and boost computational efficiency. Its learning rate scheduling uses a warm-up cosine strategy that is dynamically updated per iteration. At each update of the student model parameters, there is an update of the teacher model using EMA.

This paper uses the BCE loss function [28] and Dice loss function [29] to train the model in handling image segmentation problems with imbalanced categories. The specific formulas are as follows:

(16) $$L=\omega (t)\cdot L_{cons}\;+L_{\mathrm{BCE}}+L_{\mathrm{Dice}}+\lambda_{m}L_{Mahalanobis}$$

(17) $$L_{\mathrm{BCE}}=-\frac{1}{N}\sum_{i=1}^{N}\left[G_{i}\ln P_{i}+(1-G_{i})\ln(1-P_{i})\right]$$

(18) $$L_{\mathrm{Dice}}=1-\frac{2\sum_{i=1}^{N}G_{i}P_{i}}{\sum_{i=1}^{N}G_{i}^{2}+\sum_{i=1}^{N}P_{i}^{2}}$$

(19) $$L_{cons}=\sum p_{s}\log\frac{p_{s}}{p_{t}}$$

### 4.4. Comparative Experiments

This section validates the effectiveness of the proposed method by comparing it with U-Net [12], U-Net++ [13], CFANet [16], ResNet-UNet [15], PraNet [17], and CSCAU-Net [18]. Each model is trained and tested on the same dataset with identical training parameters. Quantitative evaluation is based on Dice, Accuracy, Recall and MIoU.

**Figure 5:**
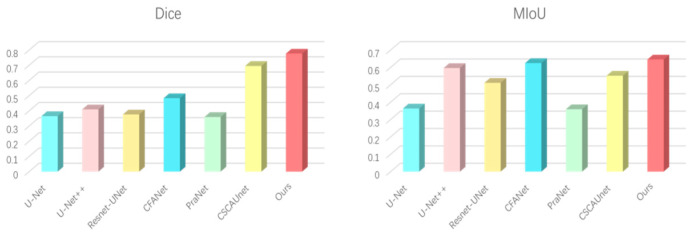
Statistics of Dice and MIoU metrics for each model.

As illustrated in Table 1 and Figure 5, the experimental results reveal that most evaluation metrics can achieve optimal performance using MSAF-Net. In comparison with the baseline U-Net, ResNet’s performance is similar on both metrics, with Dice scores of 0.428 and 0.376, respectively, while MIoU remains at 0.494 and 0.511. These results indicate that the models have difficulty handling complex pathological structures. Our methods obtained Dice scores approximately 0.118 higher and MIoU approximately 0.018 higher compared to CFANet, reflecting the advantage of MSAF-Net for enhancing feature representation. TransUNet achieves a Dice score of 0.699. This result may be attributed to its ability to combine global contextual information from Transformers with local feature representations from U-Net.

The results indicate that the proposed method can better distinguish lesion regions from surrounding tissues. The improvement is related to the introduction of the MDPF and DCA modules. MDPF extracts features at different scales and helps retain more lesion information. DCA strengthens important feature responses during decoding and reduces the influence of irrelevant information. These improvements help the network generate more accurate segmentation results. The performance gain is also related to the semi-supervised training strategy. Unlabeled samples provide additional information during training. This helps improve feature learning and refines model robustness under limited annotation conditions.

**Figure 6:**
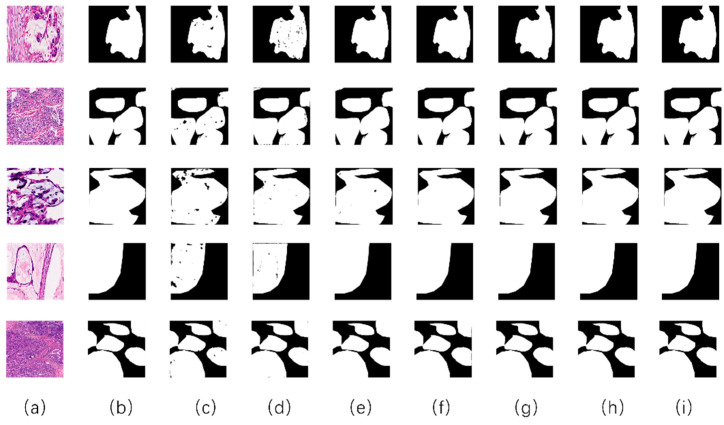
Visualization results of the MSAF-Net comparison experiment on SEED-Gastric Carcinoma stage 1 (**a**) Input image; (**b**) Ground truth; (**c**) U-Net; (**d**) U-Net++; (**e**) ResNet-UNet; (**f**) CFANet; (**g**) PraNet; (**h**) CSCAU-Net; (**i**) Ours. The color images represent the original histopathological input images, and the black-and-white images show the ground truth and segmentation results. The white regions indicate the segmented foreground regions, while the black regions indicate the background.

The results of visualization are demonstrated in Figure 6. Most comparison models have different levels of interference from background structure during segmentation, often misclassifying non-lesion regions as foreground. By contrast, the proposed MSAF-Net better suppresses the background noise and provides a more concentrated and continuous response to the lesion regions, which leads to more precise boundary predictions and reduces structural errors due to the misclassification of background. Some comparison methods generate discontinuous prediction regions and miss part of the lesion structures. In contrast, MSAF-Net produces more complete segmentation results and preserves the overall lesion shape more effectively. Similar findings can also be observed from the quantitative results in Table 1.

### 4.5. Statistical Validation

As shown in Table 2, MSAF-Net consistently outperforms U-Net on all evaluation metrics. The Dice score improves from 0.428 ± 0.053 to 0.776 ± 0.011. The MIoU also rises from 0.494 ± 0.063 to 0.645 ± 0.019. In addition, both Accuracy and Recall obtain higher values. Statistical tests reveal that the differences are statistically significant (*p* < 0.05).

### 4.6. Ablation Experiments

In this section, ablation experiments are conducted systematically to investigate the contribution of each module in the U-Net backbone. Based on the baseline U-Net, we gradually integrate the MDPF module, enhanced loss function, and DCA module.

As presented in Table 3, with the help of the MDPF module, the Dice score increased from 0.428 to 0.644, and the MIoU rose from 0.494 to 0.502. The results indicate that the MDPF module can integrate feature information at different scales and alleviate the problem of insufficient information under single-scale features. Thus, it enriches the representational capabilities of the network.

Based on this, we introduce DMC Loss to guide model training through category-centric constraints. As illustrated in Table 3, MSAF-Net further improves Dice and MIoU to 0.659 Dice and 0.595 MIoU. It is also verified that the additional loss can suppress background interference effectively, which helps the model pay more intense attention to the critical lesion area.

Finally, the decoder was replaced with the DCA module. MSAF-Net achieved the best results on the stage 1 dataset: Dice reached 0.776, and MIoU reached 0.645. It can be attributed to the fact that the DCA module refines the decoder in the characterization of lesion edges and contours by modeling channel and spatial information due to attention mechanisms and guides the decoder to pay more attention to the critical restoration of regions.

## 5. Conclusions and Discussion

This study presents a U-Net-based semi-supervised attention network for gastric cancer detection. MSAF-Net overcomes the problems brought about by small targets, weak boundaries, and the high cost of annotation in gastric cancer pathology images. In this research, several improvements were carried out on the basic U-Net architecture, such as the MDPF module, the DCA module, and the DMC Loss based on the Mahalanobis distance. The MDPF module enriches multiscale feature expression, which are important for identifying complex glandular patterns. On the other hand, the DCA module enhances decoding by strengthening key region reconstruction, allowing for improved detail restoration of blurred structures. The Mahalanobis distance loss function is effective in distinguishing the tumor region from the surrounding area.

In summary, the proposed method provides an effective framework for gastric cancer pathology image segmentation. Through the enhancement of detecting tumor areas and morphologies, this framework may help pathologists in their quantitative analysis and potentially aid in computer-aided diagnosis systems.

Several limitations remain in the current study. The Mean Teacher framework requires an additional teacher network during training, which increases training time and memory consumption. In addition, the experiments were conducted on the SEED-Gastric Carcinoma stage 1 dataset only. The influence of severe slide artifacts, tissue folds, and low-quality pathological images was not separately evaluated. These factors may affect model performance in practical applications.

Additional pathological datasets will be included in future studies. Cases with slide artifacts and poor image quality will be examined in greater detail. Further efforts will also be made to reduce the computational cost of the framework.

## Figures and Tables

**Table 1:** Performance comparison of our method with other advanced methods.

Method	Dice	Accuracy	Recall	MIoU
U-Net	0.428	0.762	0.614	0.494
U-Net++	0.436	0.770	0.773	0.597
ResNet-UNet	0.376	0.742	0.645	0.511
CFANet	0.658	0.813	0.752	0.627
PraNet	0.359	0.718	0.500	0.359
CSCAU-Net	0.695	0.764	0.688	0.552
TransUNet	0.699	0.770	0.689	0.556
Ours	0.776	0.821	0.772	0.645

**Table 2:** Statistical comparison between U-Net and MSAF-Net.

Method	Dice	Accuracy	Recall	MIoU
U-Net	0.428 ± 0.053	0.762 ± 0.027	0.614 ± 0.056	0.494 ± 0.063
Ours	0.776 ± 0.011	0.821 ± 0.026	0.772 ± 0.018	0.645 ± 0.019

**Table 3:** Ablation experiments of the MSAF-Net.

Description				Metrics			
Backbone (U-Net)	MDPF	DMC	DCA	Dice	Accuracy	Recall	MIoU
✓				0.428	0.762	0.614	0.494
✓	✓			0.644	0.793	0.635	0.502
✓	✓	✓		0.659	0.799	0.717	0.595
✓	✓	✓	✓	0.776	0.821	0.772	0.645

Note: Check marks indicate the modules included in each ablation setting.

## Data Availability

The dataset used in this study is publicly available from the original data source. Further information can be obtained from the corresponding author upon reasonable request.
